# Metabotropic glutamate receptor genetic variants and peripheral receptor expression affects trait scores of autistic probands

**DOI:** 10.1038/s41598-024-59290-2

**Published:** 2024-04-12

**Authors:** Nilanjana Dutta, Mahasweta Chatterjee, Sharmistha Saha, Swagata Sinha, Kanchan Mukhopadhyay

**Affiliations:** Manovikas Biomedical Research and Diagnostic Centre, Manovikas Kendra, 482 Madudah, Plot I-24, Sector J, EM Bypass, Kolkata, West Bengal 700107 India

**Keywords:** Biotechnology, Genetics, Molecular biology, Psychology, Biomarkers, Medical research, Molecular medicine

## Abstract

Glutamate (Glu) is important for memory and learning. Hence, Glu imbalance is speculated to affect autism spectrum disorder (ASD) pathophysiology. The action of Glu is mediated through receptors and we analyzed four metabotropic Glu receptors (mGluR/GRM) in Indo-Caucasoid families with ASD probands and controls. The trait scores of the ASD probands were assessed using the Childhood Autism Rating Scale2-ST. Peripheral blood was collected, genomic DNA isolated, and GRM5 rs905646, GRM6 rs762724 & rs2067011, and GRM7 rs3792452 were analyzed by PCR/RFLP or Taqman assay. Expression of mGluRs was measured in the peripheral blood by qPCR. Significantly higher frequencies of rs2067011 ‘A’ allele/ AA’ genotype were detected in the probands. rs905646 ‘A ‘exhibited significantly higher parental transmission. Genetic variants showed independent as well as interactive effects in the probands. Receptor expression was down-regulated in the probands, especially in the presence of rs905646 ‘AA’, rs762724 ‘TT’, rs2067011 ‘GG’, and rs3792452 ‘CC’. Trait scores were higher in the presence of rs762724 ‘T’ and rs2067011 ‘G’. Therefore, in the presence of risk genetic variants, down-regulated mGluR expression may increase autistic trait scores. Since our investigation was confined to the peripheral system, in-depth exploration involving peripheral as well as central nervous systems may validate our observation.

## Introduction

The key features of autism spectrum disorder (ASD), a group of neurodevelopmental disorders, are persistent deficits in social communication and social interaction skills along with restricted, repetitive patterns of behavior, interests, or activities^1^. Various other traits including hyperactivity, sensory dysregulation, intellectual deficit, and oppositional defiant disorder are also detected often as co-morbid features^2^. In the US, about 1 in 54 children aged 8 years were found to be affected with ASD, indicating a prevalence of 1.85%^3^. A male biasness (about 4 times higher in males than females) was also observed^3^. In the Indian children, the prevalence was reported to be 0.23% in Kerala, southern India^4^, and Kolkata, eastern India^5^.

The behavioral abnormalities associated with ASD were speculated to occur due to an imbalance between the excitatory and inhibitory neurotransmitters^6^. Abnormal signalling of glutamate (Glu), the principal excitatory neurotransmitter in the central as well as peripheral nervous systems was reported in autistic subjects^7,8^. The action of Glu is meditated through the Glu receptors (GluR) distributed widely in the cerebellum and hippocampus, the brain regions targeted for studying the etiology of ASD^9^. The cerebellum regulates motor activity, attention, cognitive functions, and sensory sensitivities, traits that are impaired in autistic individuals^10^. Moreover, the glutamatergic signalling mediated by the GluR was found to have a major role in the developing cortex^11^.

GluRs fall into two principal categories, ionotropic (iGluRs) and metabotropic (mGluRs). The mGluRs are detected in the pre- and postsynaptic neurons of the hippocampus, cerebellum^12^, and cerebral cortex, as well as other parts of the brain and peripheral tissues^13^. They are involved in learning, memory, anxiety, and the perception of pain^14^. Based on the receptor structure and physiological activity^14^, mGluRs are labelled as mGluR_1_ to mGluR_8_ (GRM1 to GRM8). Alterations in genes encoding for mGluR6^15^ and mGluR7^16^ were reported to be associated with ASD in the French and Chinese populations respectively. Experimental mouse models, with heterozygous or homozygous null mutations in the gene encoding for mGluR5, exhibited altered motor and social behaviors^17^. However, the association between mGluR and ASD has not yet been explored in the Indian population. In this pioneering investigation, we have studied the relationship between ASD and genes coding for mGluR 5–7 (GRM5, GRM6, and GRM7) in the Indo-Caucasoid population^18^.

## Results

### Case–control comparative analysis

Genotypes of all four markers followed the Hardy–Weinberg Equilibrium (HWE) both in the case and control groups (*P* > 0.05). Comparative analysis (Table 1) showed significantly higher frequency of rs2067011 ‘A’ allele (*P* < 0.0001; Power = 96%; OR 1.59) and ‘AA’ genotype (*P* < 0.0001; Power = 99%, OR 1.54) in the ASD probands. Gender-based stratified analysis (Table 1) showed a significantly higher frequency of rs2067011 ‘A’ allele (*P* = 0.001; Power = 80%; OR 1.58) and rs2067011 ‘AA’ genotype (*P* = 0.002; Power = 87%; OR 4.99) in the male probands. The female probands also showed a higher frequency of rs2067011 ‘A’ allele (*P* = 0.04; Power = 41%; OR 1.52) and rs2067011 ‘AA’ genotype (*P* = 0.03; Power = 62%; OR 1.36). No statistically significant differences in allelic and genotypic frequencies were detected for rs905646, rs762724, and rs3792452 (Table 1).

**Table 1:** Case–control comparative analysis on the studied GluR genetic variants.

Variant	Allele/ Genotype	Controls	ASD Probands	χ2 (P)	Odds Ratio (OR) 95% Confidence Interval (95% CI)	Male control	Male probands	χ2 (P)	Odds Ratio (OR) 95% Confidence Interval (95% CI)	Female control	Female probands	χ2 (P)	Odds Ratio (OR) 95% Confidence Interval (95% CI)
rs905646	G	0.15	0.14	0.16 (0.68)	0.94 (0.69–1.26)	0.14	0.14	0.05 (0.80)	1.04 (0.71–1.54)	0.16	0.13	0.68 (0.40)	0.78 (0.44–1.39)
	A	0.85	0.86		1.06 (0.78–1.43)	0.86	0.86		0.95 (0.64–1.40)	0.84	0.87		1.26 (0.71–2.24)
	GG	0.02	0.01	0.57 (0.74)	0.66 (0.23–1.92)	0.02	0.02	0.06 (0.96)	1.85 (0.45–7.52)	0.03	0	3.12 (0.20)	0.26 (0.03–1.85)
	GA	0.25	0.25		1.00 (0.71–1.41)	0.24	0.25		1.03 (0.66–1.61)	0.26	0.25		0.99 (0.51–1.92)
	AA	0.72	0.73		2.69 (2.03–3.55)	0.74	0.73		0.95 (0.61–1.46)	0.71	0.75		1.16 (0.61–2.22)
rs762724	C	0.48	0.45	0.72 (0.39)	0.90 (0.72–1.13)	0.49	0.46	0.82 (0.36)	0.87 (0.65–1.17)	0.47	0.45	0.14 (0.70)	0.91 (0.58–1.43)
	T	0.52	0.55		1.10 (0.87–1.38)	0.51	0.54		1.14 (0.85–1.53)	0.53	0.55		1.08 (0.69–1.70)
	CC	0.20	0.18	0.80 (0.66)	0.86 (0.57–1.29)	0.22	0.18	1.27 (0.52)	0.74 (0.43–1.24)	0.19	0.20	0.95 (0.62)	1.10 (0.49–2.46)
	CT	0.55	0.55		0.98 (0.71–1.35)	0.53	0.56		1.11 (0.73–1.69)	0.56	0.49		0.74 (0.39–1.39)
	TT	0.25	0.27		1.14 (0.79–1.65)	0.24	0.27		1.11 (0.69–1.78)	0.25	0.31		1.35 (0.66–2.77)
rs2067011	A	0.54	0.65	17.27 **(<** **0.0001)**	1.59 (1.27–1.98)	0.54	0.65	9.73 **(0.001)**	1.58 (1.18–2.11)	0.53	0.64	3.97 **(0.04)**	1.52 (1.00–2.31)
	G	0.46	0.35		0.62 (0.50–0.78)	0.46	0.35		0.63 (0.47–0.84)	0.47	0.36		0.65 (0.43–0.99)
	AA	0.30	0.40	21.68 **(<** **0.0001)**	1.54 (1.12–2.12)	0.30	0.40	11.80 **(0.002)**	4.99 (3.10–8.04)	0.30	0.36	6.58 **(0.03)**	1.36 (0.72–2.56)
	AG	0.48	0.51		1.11 (0.82–1.51)	0.48	0.50		1.02 (0.68–1.51)	0.48	0.56		1.34 (0.75–2.42)
	GG	0.22	0.09		0.39 (0.26–0.60)	0.22	0.10		0.38 (0.21–0.68)	0.22	0.08		0.40 (0.19–0.85)
rs3792452	C	0.88	0.86	0.60 (0.43)	0.87 (0.63–1.21)	0.90	0.86	3.05 (0.08)	0.68 (0.45–1.05)	0.86	0.88	0.28 (0.59)	1.18 (0.63–2.19)
	T	0.12	0.14		1.13 (0.82–1.56)	0.10	0.14		1.44 (0.95–2.20)	0.14	0.12		0.84 (0.45–1.57)
	CC	0.78	0.76	0.55 (0.75)	0.88 (0.61–1.26)	0.82	0.76	2.78 (0.24)	6.08 (3.13–11.80)	0.74	0.75	2.90 (0.23)	1.05 (0.52–2.10)
	CT	0.20	0.21		1.01 (0.75–1.61)	0.16	0.20		1.31 (0.79–2.16)	0.23	0.25		1.12 (0.54–2.30)
	TT	0.02	0.03		1.26 (0.49–3.24)	0.02	0.04		1.98 (0.62–6.38)	0.03	0		0.27 (0.03–2.00)

Significant values are in bold.

### Family-based analysis of allelic transmission

Biased parental transmissions of rs905646 ‘A’ allele to all probands (*P* = 0.01; Power = 67%) and the male probands (*P* = 0.006; Power = 77%) were detected (Table 2). The gender-based stratified analysis revealed paternal over-transmission of rs905646 ‘A’ allele to all probands (*P* = 0.04; Power = 85%) and the male probands (*P* = 0.01; Power = 97%). No significant bias in the transmission pattern was observed for rs762724, rs2067011, and rs3792452 (Supplementary Table S1). Analysis of families with male- and female-only probands also failed to show any gender-specific effect (Supplementary Table S1).

**Table 2:** Analysis of allelic transmission in families with ASD probands by Transmission Disequilibrium Test.

Variant	Parent Group	Probands	Allele	Transmitted (T)	Non-Transmitted (NT)	χ2 (P)
rs905646	Both	All probands	G	0.13	0.20	5.81 **(0.01)**
			A	0.87	0.80	
		Male probands	G	0.12	0.21	7.28 **(0.006)**
			A	0.88	0.79	
		Female probands	G	0.15	0.13	0.07 (0.78)
			A	0.85	0.87	
	Father	All probands	G	0.13	0.19	4.17 **(0.04)**
			A	0.87	0.81	
		Male probands	G	0.13	0.22	6.007 **(0.01)**
			A	0.87	0.78	
		Female probands	G	0.13	0.09	0.31 (0.57)
			A	0.87	0.91	
	Mother	All probands	G	0.13	0.18	2.85 (0.09)
			A	0.87	0.82	
		Male probands	G	0.13	0.19	3.04 (0.08)
			A	0.87	0.81	
		Female probands	G	0.13	0.14	0.01 (0.89)
			A	0.87	0.86	

Significant values are in bold.

### Comparative analysis on haplotype frequencies

A significantly higher occurrence of rs762724-rs2067011 ‘T-A’ haplotype (Table 3; *P* < 0.0001, OR 3.38, Power = 99%) was observed in the ASD probands, which remained significant even after gender-based stratified analysis in the male probands (*P* < 0.0001, OR 3.26, Power = 98%) and in the female probands (*P* < 0.0001, OR 4.26, Power = 99%). This was concomitant with a lower occurrence of the ‘T-G’ haplotype in the probands (*P* < 0.0001, OR 0.53, Power = 97%), male probands (*P* = 0.0001, OR 0.51, Power = 67%), and female probands (*P* = 0.02, OR 0.57, Power = 67%) (Table 3).

**Table 3:** Population-based comparative analysis on haplotype frequency.

Marker combination	Proband Groups	Haplotypes	Control	Probands	Odds Ratio (OR) 95% Confidence Interval (95% CI)	χ2 (P value)
rs762724- rs2067011	All	C-A	0.43	0.40	0.88 (0.70–1.11)	0.27 (0.60)
		C-G	0.05	0.05	1.08 (0.65–1.81)	0.77 (0.37)
		T-A	0.09	0.26	3.38 (2.49–4.58)	55.45 **(<** **0.0001)**
		T-G	0.43	0.29	0.53 (0.42–0.67)	22.78 **(<** **0.0001)**
	Male	C-A	0.43	0.40	0.86 (0.63–1.15)	0.27 (0.59)
		C-G	0.06	0.06	1.03 (0.54–1.95)	1.05 (0.30)
		T-A	0.08	0.26	3.26 (2.24–4.75)	34.08 **(<** **0.0001)**
		T-G	0.43	0.28	0.51 (0.38–0.70)	14.11 **(0.0001)**
	Female	C-A	0.43	0.41	0.93 (0.59–1.46)	0.02 (0.87)
		C-G	0.05	0.04	0.88 (0.29–2.60)	0.32 (0.56)
		T-A	0.10	0.25	4.26 (2.19–8.31)	15.43 **(<** **0.0001)**
		T-G	0.43	0.30	0.57 (0.36–0.90)	5.25 **(0.02)**

Significant values are in bold.

### Multifactor dimensionality reduction (MDR) analysis

**Figure 1:**
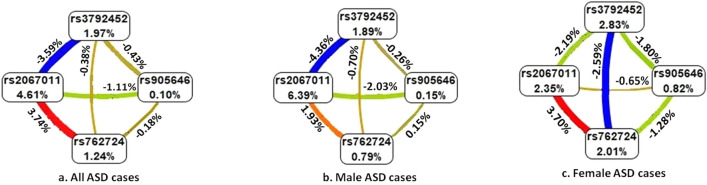
Multifactor Dimensionality Reduction (the entropy model) analysis was performed to study the interaction between the studied gene variants: (**a**) All ASD cases; (**b**) Male ASD cases; (**c**) Female ASD cases.

Analysis of the case–control dataset revealed independent effect of rs905646 (Information Gain; IG = 0.10%), rs762724 (IG = 1.24%), rs2067011 (IG = 4.61%), and rs3792452 (IG = 1.97%), along with synergistic interactions between rs762724-rs2067011 (IG = 3.74%) in the ASD probands (Fig. 1a). In the male probands, rs905646 (IG = 0.15%), rs762724 (IG = 0.79%), rs2067011 (IG = 6.39%), and rs3792452 (IG = 1.89%) showed independent effects (Fig. 1b), while rs762724-rs2067011 (IG = 1.93%) showed moderate synergistic effects as compared to the gender-matched controls (Fig. 1b). In the case of the female ASD probands, MDR analysis revealed independent effects of rs905646 (IG = 0.82%), rs762724 (IG = 2.01%), rs2067011 (IG = 2.35%), and rs3792452 (IG = 2.83%), with synergistic interactions between rs762724-rs2067011 (IG = 3.70%) (Fig. 1c).

### Linkage disequilibrium (LD) analysis between the pair of markers

Strong LD was detected between rs762724-rs2067011 in the control group (Supplementary Fig. 1a; D' = 0.78, r^2^ = 0.51) as compared to the ASD probands (Supplementary Fig. 1a; D' = 0.64, r^2^ = 0.18). The gender-based stratified analysis also revealed strong LD in the male control (Supplementary Fig. 1c; D' = 0.76, r^2^ = 0.53) and female control (Supplementary Fig. 1e; D' = 0.79, r^2^ = 0.51) groups as compared to the probands groups (Supplementary Fig. 1d and f.).

### Quantitative trait (QT) analysis

Genotype–phenotype association analysis (Table 4) revealed lower scores for Emotional response in the presence of rs762724 ‘CC’ genotype (*P* = 0.01). Scores were higher in the presence of the ‘T’ allele, though the difference was statistically insignificant ((*P* > 0.05). In the presence of rs2067011 ‘G’ variant, scores for the Listening response and Visual response were higher (*P* > 0.02). The trait Object use was found to be positively and negatively influenced by rs3792452 ‘TT’ (*P* = 0.04) and rs905646 ‘GG’ (*P* = 0.04) respectively.

**Table 4:** Quantitative Trait analysis to identify association between the studied genetic variants and autistic traits.

Proband	Trait	Variant	Allele/Genotype	Add Value (AddVal)	χ^2^ (P)
All	Emotional response	rs762724	CC	− 1.00	**5.77** **(0.01)**
			CT	1.04	2.47 (0.11)
			TT	0.92	0.12 (0.72)
	Listening response	rs2067011	A	− 0.46	4.84 **(0.02)**
			G	0.46	
			AA	− 0.43	2.14 (0.14)
			AG	0.24	0.008 (0.92)
			GG	1.35	6.49 **(0.01)**
	Visual response		A	− 0.62	7.86 **(0.005)**
			G	0.62	
			AA	− 0.76	6.10 **(0.01)**
			AG	0.62	1.14 (0.28)
			GG	1.48	5.09 **(0.02)**
	Object use	rs3792452	CC	0.05	0.03 (0.86)
			CT	− 0.24	0.89 (0.35)
			TT	2.19	3.90 **(0.04)**
		rs905646	GG	− 2.01	4.15 **(0.04)**
			GA	− 0.07	0.01 (0.90)
			AA	1.99	0.53 (0.46)
Male probands	Emotional response	rs762724	C	− 0.48	3.63 **(0.05)**
			T	0.48	
			CC	− 1.31	7.18 **(0.007)**
			CT	1.31	1.98 (0.15)
			TT	1.31	0.54 (0.46)
	Listening response	rs2067011	A	− 0.54	5.69 **(0.01)**
			G	0.54	
			AA	− 0.43	1.84 (0.17)
			AG	0.17	0.29 (0.58)
			GG	1.62	9.38 **(0.002)**
	Visual response		A	− 0.61	6.39 **(0.01)**
			G	0.61	
			AA	− 0.70	4.24 **(0.03)**
			AG	0.53	0.40 (0.52)
			GG	1.49	5.07 **(0.02)**
	Object use	rs3792452	CC	− 0.28	0.69 (0.40)
			CT	0.06	2.758e−005(0.99)
			TT	2.35	4.26 **(0.03)**
		rs905646	GG	− 2.03	3.79 **(0.05)**
			GA	− 0.18	0.16 (0.68)
			AA	0.32	1.09 (0.29)
	Body use	rs762724	CC	− 0.76	4.85 **(0.02)**
			CT	0.75	1.14 (0.28)
			TT	0.78	0.50 (0.47)
Female probands	Non-verbal communication	rs762724	C	− 1.23	5.98 **(0.01)**
			T	1.23	
			CC	− 1.65	2.72 (0.09)
			CT	1.21	0.36 (0.54)
			TT	2.74	5.25 **(0.02)**
	Verbal communication		C	− 1.33	7.54 **(0.006)**
			T	1.33	
			CC	− 1.51	3.01 (0.08)
			CT	0.97	0.79 (0.37)
			TT	3.13	7.32 **(0.006)**
		rs2067011	A	− 0.90	3.86 **(0.04)**
			G	0.90	
			AA	− 1.42	4.27 **(0.03)**
			AG	1.32	1.91 (0.16)
			GG	2.19	1.40 (0.23)
		rs3792452	C	1.83	5.22 **(0.02)**
			T	− 1.83	
			CC	2.12	5.97 **(0.01)**
			CT	− 2.12	
	Fear or nervousness	rs762724	CC	− 0.44	0.20 (0.64)
			CT	− 0.39	2.94 (0.08)
			TT	2.20	5.84 **(0.01)**
	Adaptation to change	rs2067011	A	− 0.82	4.40 **(0.03)**
			G	0.82	
			AA	− 1.20	4.59 **(0.03)**
			AG	1.1	1.91 (0.16)
			GG	2.12	1.76 (0.18)
	Object use	rs3792452	C	1.26	3.94 **(0.04)**
			T	− 1.26	
			CC	1.48	4.47 **(0.03)**
			CT	− 1.48	4.47 **(0.03)**
	Emotional response	rs905646	G	− 1.75	5.26 **(0.02)**
			A	1.75	
			GA	− 2.10	6.17 **(0.01)**
			AA	2.10	
	Relating to people		G	− 1.58	3.61 **(0.05)**
			A	1.58	
			GA	− 1.88	4.31 **(0.03)**
			AA	1.88	
		rs3792452	CC	1.99	3.82 **(0.05)**
			CT	− 1.99	
	Level and consistency of intellectual response	rs2067011	AA	− 1.03	1.90 (0.16)
			AG	1.31	3.86 **(0.04)**
			GG	− 1.04	1.34 (0.24)
	Total CARS	rs3792452	C	0.19	3.73 **(0.05)**
			T	− 0.19	
			CC	0.22	4.27 **(0.03)**
			CT	− 0.22	

Significant values are in bold.

The gender-based stratified analysis also showed a similar trend in the male probands for rs762724, rs2067011, rs3792452, and rs905646 (Table 4). Additionally, the score for Body use was lower in the presence of rs762724 ‘CC’ in the male probands (*P* = 0.02).

In the female probands, in the presence of rs762724 ‘T’ scores for Non-verbal (*P* > 0.02) and Verbal communications (*P* > 0.006), as well as Fear or nervousness (*P* = 0.01) were increased (Table 4). rs3792452 ‘C’ also affected the Verbal communication positively (*P* > 0.02). On the other hand, scores for Object use, Relating to people, and Total CARS were lower in the presence of rs3792452 ‘T’ allele (*P* > 0.05). rs2067011 ‘A’ negatively affected the scores for Verbal communication, Adaptation to change, and Level and consistency of intellectual response. Scores for Emotional response and Relating to people were higher in the presence of rs905646 ‘A’.

### GluR mRNA expression

**Figure 2:**
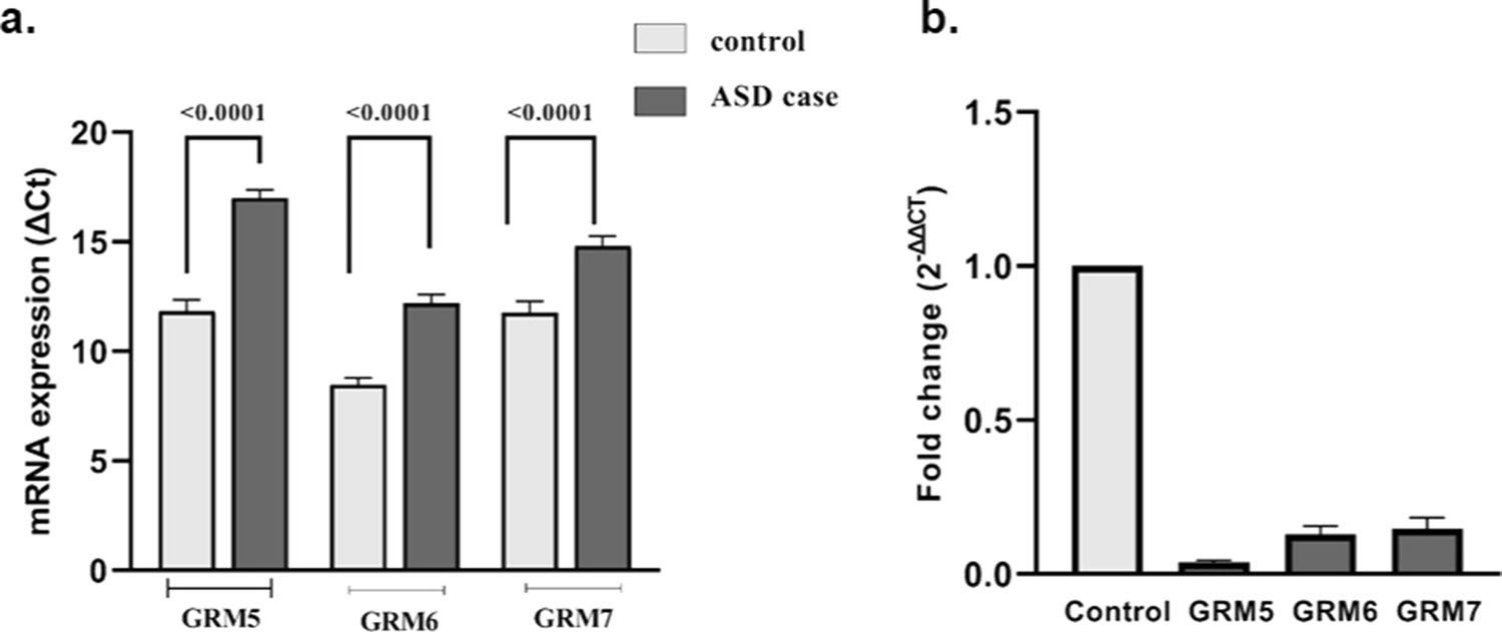
Relative mRNA expression of the glutamatergic genes in the peripheral blood: (**a**) Comparative analysis of normalized GRM5, GRM6, and GRM7 expression (ΔCt) in ASD cases and age-matched controls; (**b**) relative fold change in the expression of these genes in ASD cases.

Case–control comparative analysis showed **s**tatistically significant lower expressions of GRM5, GRM6, and GRM7 mRNA in the probands as compared to the age-matched controls (Fig. 2a; *P* < 0.0001). Analysis of relative mRNA expression showed several-fold-down regulations for the GRM5 (25), GRM6 (8.33), and GRM7 (7.14) in the probands as compared to the controls (Fig. 2b).

**Figure 3:**
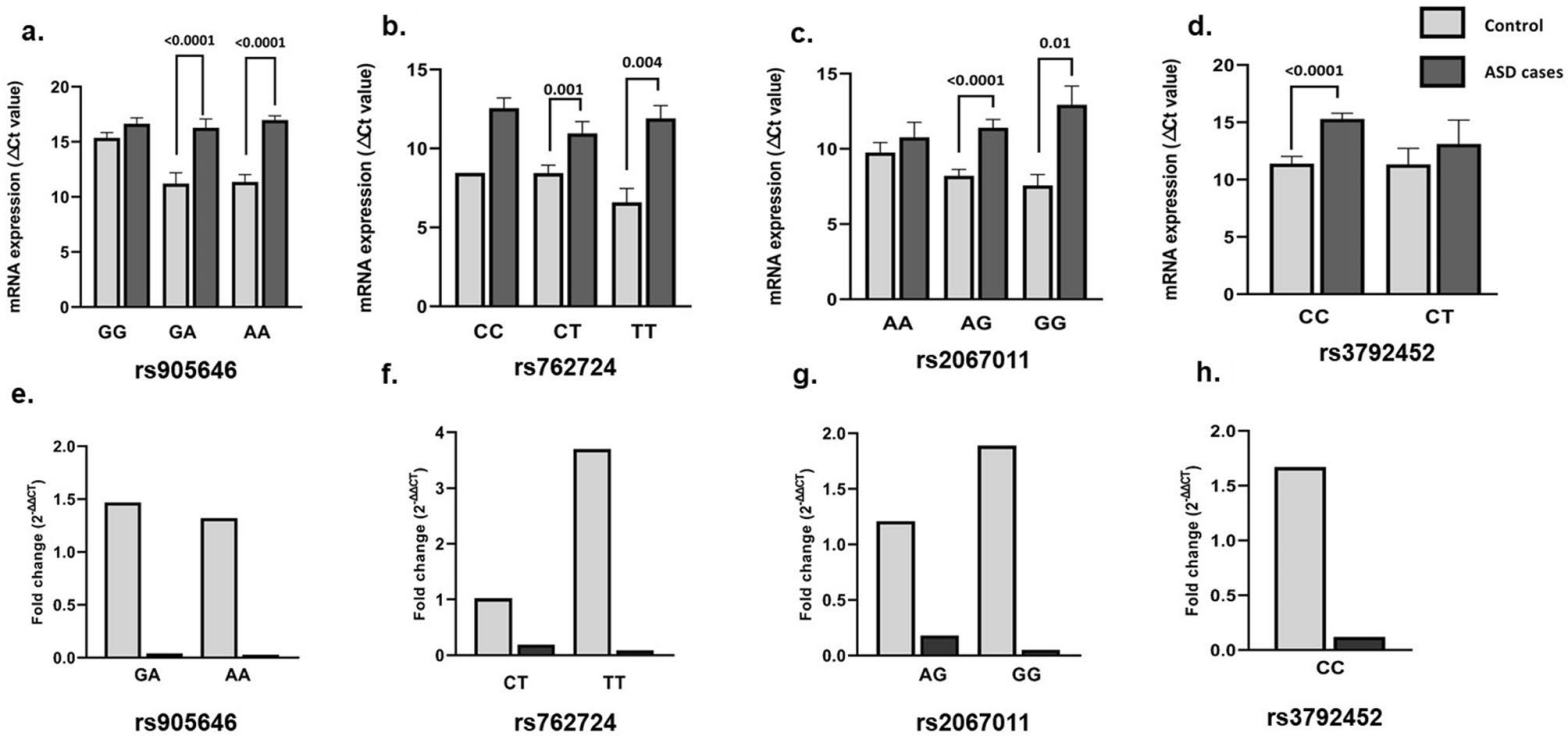
Genotype-based stratified analysis on the expression of GRM5, GRM6, and GRM7 in the controls and ASD probands having different genotypes: (**a**) rs905646; (**b**) rs762724; (**c**) rs2067011; (**d**) rs3792452.

Stratified analysis revealed significantly lower expression in the ASD probands carrying GRM5 rs905646 ‘GA’ and ‘AA’ (Fig. 3a; *P* < 0.001), GRM6 rs762724 ‘CT’ and ‘TT’ (Fig. 3b; *P* < 0.004), GRM6 rs2067011 ‘AG’ and ‘GG’ (Fig. 3c P < 0.01), and GRM7 rs3792452 ‘CC’ (Fig. 3d; *P* < 0.0001) genotypes as compared to controls. Comparative analysis on normalized gene expression showed down regulated GRM5 expression (Fig. 3e–h) in ASD probands having rs905646 ‘GA’ (25-fold) and ‘AA’ (33.33-fold), down regulated GRM6 expression in the presence of rs762724 ‘CT’ (5.2) and ‘TT’ (11-fold), rs2067011 ‘AG’ (5.5 fold) and ‘GG’ (20-fold), as well as down regulated GRM7 expression (8.3-fold) in the probands carrying rs3792452 ‘CC’ genotypes, as compared to controls having the same variants.

## Discussion

The present investigation on the Indo-Caucasoid ASD probands revealed that in the presence of genetic variants, metabotropic glutamate receptor mRNA expression is significantly down-regulated which may lead to the severity of ASD traits.

The GRM5 gene, located on chromosome 11, encodes for a mGluR whose signaling activates a second messenger system by phosphatidylinositol-calcium^19^. The role of GRM5 has been implicated in various neurological disorders including ASD^17^ and obsessive–compulsive disorder^20^. GRM5 knock-out rats exhibited autistic symptoms^21^. Investigators also reported decreased mGluR5 expression in the dorsolateral prefrontal cortex of ASD probands^22^. On the other hand, a positron emission tomography study revealed higher expression of GRM5 in the cortical region of high-functioning ASD probands; the investigators speculated that this could be a molecular feature associated with superior intelligence^23^. Increased mGluR5 levels were also detected in the superior frontal cortex^24^, post-central gyrus and cerebellum^25^, and left striatum/thalamus^26^ of autistic individuals. Genetic association analysis on Caucasian ASD subjects revealed a protective role of GRM5 rs905646^27^. On the other hand, our pilot investigation on the Indo-Caucasoid ASD probands documented higher parental transmission of rs905646 ‘A’ variant, as well as increased trait scores and down-regulated peripheral GRM5 expression in the presence of rs905646 ‘A’ allele. Based on the data obtained in the present study it can be speculated that GRM5 rs905646 A may be a risk factor for ASD which merits further investigation.

The GRM6 variants, rs762724 and rs2067011, were reported to have an association with higher myopia in the Han Chinese population^28^. The present study on the Indian ASD probands for the first time revealed higher trait scores for Non-verbal as well as Verbal communication, and Fear or nervousness in female ASD probands harboring the rs762724 ‘T’ allele. On the other hand, the ASD probands and male probands exhibited lower scores for Emotional response and Body use respectively in the presence of the rs762724 ‘CC’ genotype. Additionally, probands with the rs762724 ‘T’ variant showed statistically significant downregulation in GRM6 mRNA expression. The data obtained indicates a protective role of rs762724 ‘C,’ while rs762724 ‘T’ could be considered as a risk variant for ASD which warrants further exploration in other ethnic groups.

The other GRM6 variant, rs2067011, showed a significantly higher frequency of the ‘A’ allele and ‘AA’ genotype in the Indian ASD probands. Further, while rs2067011 ‘G’ was associated with downregulated GRM6 expression, scores for several traits were lower in the presence of the ‘A’ allele.

Analysis on haplotypes revealed higher frequency of the ‘T-A’ haplotype (rs762724-rs2067011) in the proband group, which may increase the chances for down-regulated receptor expression. Additionally, while LD was strong between these two GRM6 variants in the control subjects, the proband group exhibited only mild LD, which could be due to various reasons, including the presence of mutation hotspots between the two sites residing 945 bases apart on chromosome 5. Based on these observations, a role of GRM6 can be speculated in ASD which deserves further explorative analysis.

The GRM7, widely expressed in the cerebral cortex, hippocampus, and cerebellum, was speculated to affect anxiety, fear responses, and working memory^29^. The GRM7 rs3792452 ‘C’ variant showed biased parental transmission in the Korean population^30^. Our investigation revealed a several-fold reduction in GRM7 expression in the Indo-Caucasoid ASD probands having the rs3792452 ‘CC’ genotype. Scores for Verbal communication were higher in the probands with rs3792452 ‘C’. On the other hand, rs3792452 ‘T’ showed an association with lower scores for Total CARS, Object use, and Relating to People. Our study also observed extremely low frequency of the rs3792452 ‘TT’ genotype in this ethnic group (the frequencies of occurrence in control samples, ADHD cases, ASD cases, and parents are 0.02, 0.03, 0.03 and 0.01 respectively), indicating a possible deleterious effect of the ‘T’ variant in homozygous condition. Based on the present analysis, we infer that rs3792452 may have a role in ASD which is worthy of further in-depth exploration.

Analysis of the influence of the genetic variants revealed an independent effect of all the studied mGluR genetic variants on ASD. Synergistic interactive effects of rs762724- rs2067011 were also observed.

The major weaknesses of the present study include (1) limitation in the number of female ASD probands, (2) analysis of targets only in the peripheral blood, (3) investigation of only four mGluR genetic variants, and (4) some association analyses exhibiting high power and low OR values, which could be due to type I error. This may be corrected by replication of the present study involving a larger cohort. However, this pilot study on the Indo-Caucasoid subjects’ documented substantial downregulation in mGluR expression in the presence of risk genetic variants, which could be the reason for ASD severity; the reduced receptor expression may affect the downstream signaling cascade, thereby influencing the phenotypic attributes. Our study revealed statistically significant downregulated GRM5 expression in the peripheral blood of autistic subjects. On the other hand, expression of GRM5 was reported to be enhanced in the cortical regions of a subtype of autistic individuals^23^. In murine autistic models with repetitive grooming and hyperactivity, fewer dendritic spines, reduced basal synaptic transmission, reduced frequency of miniature excitatory postsynaptic currents, and enhanced N-methyl-D-aspartate receptor-mediated excitatory currents, were also reported^31^. The authors proposed that appropriate therapies for ASD are to be carefully matched to the underlying synaptopathy phenotype^31^. Our study for the first time documented lowered expression of mGluRs in the peripheral blood of autistic individuals. Further in-depth analysis, involving the peripheral as well as central nervous system, may aid in understanding the actual role of the mGluRs in the synaptic modulation of individuals with autism.

## Methods

### Recruitment of study subjects

Individuals having gross chromosomal anomalies, Fragile-X syndrome, and other developmental or neurological disorders were excluded from the study. Nuclear families with ASD probands (N = 340), their fathers (N = 211), and mothers (N = 249) were recruited from the outpatient department of the institute following the Diagnostic and Statistical Manual of Mental Disorders criteria^1,32^. The mean age of the probands was 5.96 years ± 3.39 (Standard Deviation) and male to female ratio was 4.3:1. Recruited subjects were from the state of West Bengal, India (23°N, 87°E), belonging to the Indo-Caucasoid ethnic category^18^. Ethnically matched healthy controls (N = 396), with a male-to-female ratio of 0.86:1, and devoid of any developmental, neurological disorders, or psychiatric disorders in the family were recruited for population-based comparative analysis. All the methods were performed by the relevant guidelines and regulations and informed written consent for participation was obtained from the parents/guardians of the ASD probands and control volunteers. The study protocol was approved by the Manovikas Ethical Committee on Human Subjects, with Scientists, Psychiatrists, Psychologists, Advocates, and Social workers as members.

### Assessment of traits

In subjects with ASD, varying levels of behavioral characteristics, including (1) Relating to People, (2) Imitation, (3) Emotional response, (4) Body use, (5) Object use, (6) Adaption to change, (7) Visual response, (8) Listening response, (9) Taste, smell and touch response and use, (10) Fear or nervousness, (11) Verbal communication, (12) Nonverbal communication, (13) Activity level, (14) Level and consistency of intellectual response, and (15) General impression, are observed^33^. The severity of these 15 traits was evaluated using the Childhood Autism Rating Scale 2-Standard Test (CARS2- ST)^33^. Under this scale, the scores for each trait vary from 1 to 4 with 0.5 intervals, and subjects were categorized as mild to moderate (score 30.0–36.5) and severe (score 37.0–60.0) categories based on the total score.

### Selection and genotyping of target sites

The GRM5 (rs905646), GRM6 (rs762724 and rs2067011), and GRM7 (rs3792452) genetic variants were selected for analysis based on published reports of association with ASD or other neurodevelopmental disorders^26,27,29,34^. Peripheral blood was collected in anti-coagulant treated vials at the time of recruitment and processed for genomic DNA isolation^35^. Polymerase chain reaction in Applied Biosystems ProFlex™ followed by Restriction fragment length polymorphism (RFLP) analysis were followed for genotyping of rs905646 (G/A), rs762724 (C/T), and rs2067011 (A/G); details provided in Supplementary Table S2 and S3. Genotyping of rs3792452 (C/T) was performed in Quanto Studio3, Thermo Fisher Scientific, using a pre-designed TaqMan genotyping assay (Assay ID C_27483793_20).

### Messenger ribonucleic acid (mRNA) expression analysis

The mRNA expressions for GRM5, GRM6, and GRM7 were examined in ASD cases (N = 50) and age-matched control subjects (N = 41); details of primer sequences used are provided in Supplementary Table S4In brief, total RNA was isolated from ~ 2 ml peripheral blood using the TRIzol reagent (TRIzol Reagent User Guide; Pub.No. MAN0001271 B.0). After DNAase treatment, the RNA concentration was measured in a Qubit 4 Fluorometer and 700 ng of total RNA was reverse transcribed into complementary DNA (cDNA) using High-capacity cDNA reverse transcription kit (Applied Biosystem). Amplification was carried out in QuantStudio 3 using PowerUp SYBR Green master mix, Thermo Fisher Scientific. The cycle threshold (Ct) value for each sample was noted. The data was normalized against Glyceraldehyde 3-phosphate dehydrogenase (GAPDH) expression, serving as an endogenous control, and expressed as ΔCt. The ΔCt values for each studied genes were compared by case–control analysis. The fold change i.e. the normalized gene expression is presented as 2^−ΔΔCt^^36^ and compared with expression of these gene in the controls to understand the relative changes in gene expression.

### Statistical analyses

HWE was calculated for the studied genotypes using the online encyclopedia for genetic epidemiology studies (http://www.oege.org/software/hardy-weinberg.html). Population-based comparative analysis on allelic and genotypic frequencies was performed using the COCAPHASE program under UNPHASED (v.3.1.7) after running 1000-fold permutation tests which takes care of error for multiple testing^37^. For family-based association analysis, the Transmission Disequilibrium Test (TDT) was performed using the UNPHASED (v.3.1.7). Association between the studied genetic variants and the total CARS2-ST scores as well as independent scores for each trait was calculated using the Quantitative Trait (QT) analysis under the UNPHASED program (v.3.1.7). The odds ratio (OR) was calculated using the online program (http://www.hutchon.net/Confi dORnulhypo.html). The Power of the significant observations was calculated using Piface software (v.1.76)^38^

Pair-wise LD between the markers present on the same chromosome was analyzed using the case–control data by Haploview software (v.4.2)^39^. LD is expressed in terms of D′ where D′ is the normalized coefficient of LD and r^2^ denotes the squared correlation coefficient. Case–control comparative analysis on haplotypes was performed using the UNPHASED (v.3.1.7).

For detecting the impact of the studied genetic markers on ASD and interactions among them, the MDR (v.3.0.2) program was employed^40^. The values on the nodes IG and the connecting lines indicate the independent and interactive effects of the markers respectively. Connections in red indicate synergistic interaction between the markers while lines in blue with negative IG values indicate redundancy or lack of any synergistic interaction between the markers.

For gene expression analysis, normalized mRNA expression, relative changes, or fold change in the gene expression profile were expressed as ΔCt and 2^−ΔΔCt^ respectively. Comparative analysis of the ΔCt values and effect of studied variants on relative mRNA expression in the ASD cases and control were performed using the Mann–Whitney test under GraphPad Prism (v.9.1). Genotype based stratified analysis of ΔCt and 2^−ΔΔCt^ was also performed using the Mann–Whitney test.

### Supplementary Information

Supplementary Information.

## Data Availability

Data generated for the study are presented in tabular format as Tables, figures, and Additional files. Further details on data will be available from the corresponding author upon reasonable request.
