# Supplementary material for: Metabotropic glutamate receptor genetic variants and peripheral receptor expression affects trait scores of autistic probands
Source: Sci Rep. 2024 Apr 12;14:8558. doi: 10.1038/s41598-024-59290-2 (PMC11014995; doi:10.1038/s41598-024-59290-2)
Supplement: Supplementary file 1 — Supplementary Information. [file 41598_2024_59290_MOESM1_ESM.pdf]

1. Supplementary Tables

Table S1 Analysis of allelic transmission in families with ASD probands by Transmission Disequilibrium Test

Variant	Parent Group	Probands	Allele	T	NT	χ2(P)
rs762724	Both	All probands	C	0.45	0.42	0.45 (0.49)
			T	0.55	0.58	
		Male probands	C	0.44	0.41	0.45 (0.50)
			T	0.56	0.59	
		Female probands	C	0.47	0.45	0.02 (0.87)
			T	0.53	0.55	
	Father	All probands	C	0.44	0.44	0.01 (0.91)
			T	0.56	0.56	
		Male probands	C	0.43	0.43	0.0002 (0.98)
			T	0.57	0.57	
		Female probands	C	0.5	0.46	0.12 (0.72)
			T	0.5	0.54	
	Mother	All probands	C	0.44	0.38	1.47 (0.22)
			T	0.56	0.62	
		Male probands	C	0.43	0.37	1.25 (0.26)
			T	0.57	0.63	
		Female probands	C	0.51	0.45	0.32 (0.56)
			T	0.49	0.55	
rs2067011	Both	All probands	A	0.67	0.64	0.42 (0.51)
			G	0.33	0.36	
		Male probands	A	0.67	0.65	0.17 (0.68)
			G	0.33	0.35	
		Female probands	A	0.65	0.59	0.36 (0.54)
			G	0.35	0.41	
	Father	All probands	A	0.65	0.68	0.24 (0.62)
			G	0.35	0.32	
		Male probands	A	0.65	0.70	1.06 (0.30)
			G	0.35	0.30	
		Female probands	A	0.67	0.57	0.90 (0.34)
			G	0.33	0.43	
	Mother	All probands	A	0.67	0.64	0.26 (0.60)
			G	0.33	0.36	
		Male probands	A	0.66	0.64	0.19 (0.65)
			G	0.34	0.36	
		Female probands	A	0.67	0.65	0.06 (0.79)
			G	0.33	0.35	
rs3792452	Both	All probands	C	0.87	0.88	0.12 (0.72)
			T	0.13	0.12	
		Male probands	C	0.86	0.88	0.38 (0.53)
			T	0.14	0.12	
		Female probands	C	0.91	0.88	0.40 (0.52)
			T	0.09	0.12	
	Father	All probands	C	0.85	0.87	0.42 (0.51)
			T	0.15	0.13	
		Male probands	C	0.84	0.88	1.57 (0.20)
			T	0.16	0.11	
		Female probands	C	0.90	0.82	1.41

			T	0.10	0.18	(0.23)
	Mother	All probands	C	0.85	0.88	0.91
			T	0.15	0.12	(0.33)
		Male probands	C	0.84	0.87	0.56
			T	0.16	0.13	(0.45)
		Female probands	C	0.90	0.95	0.72
			T	0.10	0.05	(0.39)

$\chi 2$=Chi square; P=p value; RR=Relative risk; 95% CI=95% Confidence Interval.

Table S2. Details of primer sequences, reagents used in the PCR reaction, and PCR cycling conditions

Gene (Variant)	Primer sequence	Reagents of PCR reaction	PCR condition	Amplicon size (bp)
GRM5 (rs905646)	F:5'- ACATAAAGTGGGCCCAACAG -3'<br>R:5'- CATGCTGCCTGACAAAGAAT -3'	20 µl reaction mixture containing 75-100 ng of DNA, 20 pmol of each primer, 0.2 mM dNTP, 1X Taq buffer B, 1.5 mM $MgCl_2$, and 0.5 U Taq DNA polymerase	Initial denaturation at 95°C for 5 min, followed by 35 cycles of denaturation at 95°C for 30 s, annealing at 60°C for 30 s, and extension at 72°C for 30 s. Final extension at 72°C for 5 min.	242 bp
GRM6 (rs762724)	F:5'-ACAGGGAATGGTGGAACAAA-3'<br>R:5'-AATCCAGCAGGAACAGATGG-3'			361 bp
GRM6 (rs2067011)	F:5'-ACCCCCATTAGACCACTCAG-3'<br>R:5'-GGCAAGGTGCAGTTTGTGAT-3'			281 bp

Table S3. Details of RFLP-based genotyping analysis of different polymorphisms

Gene (Variant)	Restriction enzyme (Buffer)	Recognition sites	Reaction condition	Genotyping analyses	
				**Genotypes**	**Fragment sizes (bp)**
GRM5 (rs905646)	AciI (CutSmart)	5’…C▼CGC…3’<br>3’…GGC▲G…5’	37°C for 15 mins	GG<br>GA<br>AA	GG-124 and 118<br>GA-242, 124 and 118<br>AA-242
GRM6 (rs762724)	HhaI (CutSmart)	5’…CGC▼G…3’<br>3’…G▲CGC…5’	37°C for 15 mins	CC<br>CT<br>TT	CC-310 and 51<br>CT-361, 310 and 51<br>TT-361
GRM6 (rs2067011)	HpyCH4III (CutSmart)	5’…ACN▼GT…3’<br>3’…TG▲NCA…5’	37°C for 60 mins	AA<br>AG<br>GG	AA-281<br>AG-281, 159 and 122<br>GG-159 and 122

Table S4. Primer sequences used in qPCR

Gene Name	Forward primer sequence (5'-3')	Reverse primer sequence (5'-3')
GRM5	CTCAACTCCATGATGCTGTCCA	GATTTCGGCAAAGGTCGTCATG
GRM6	AGGTGCAGTTTGTGATTGATGC	ACAGCTCGAATGTACTGCAGAA
GRM7	CTCCGCGTCCTGACTTTGAT	CCCTCGATCCGGATTGAGTG

2. Supplementary Figure

Supplementary Figure. 1. Pairwise linkage disequilibrium (LD) between the studied variants analyzed using Haploview a. All control, b. All ASD probands, c. male control, d. male ASD probands, e. female control f. female ASD probands.

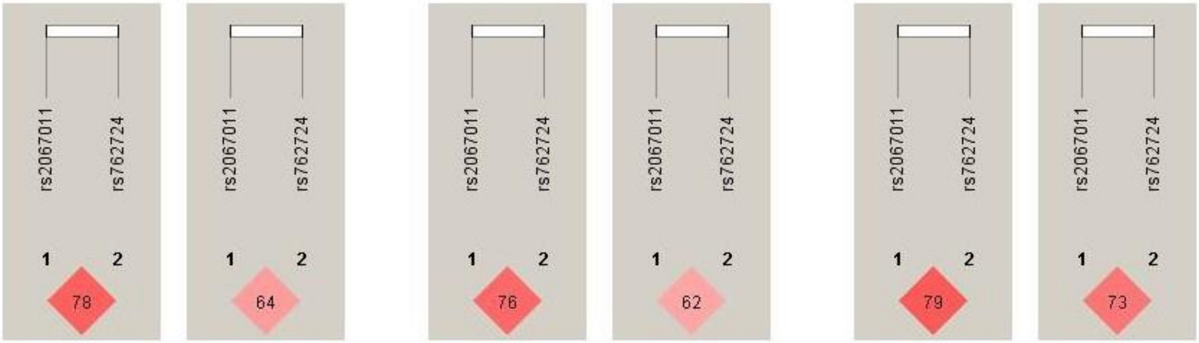

a. All controls b. All ASD cases c. Male controls d. Male ASD cases e. Female controls f. Female ASD cases
